# Antibiotic resistance in bacterial isolates from freshwater samples in Fildes Peninsula, King George Island, Antarctica

**DOI:** 10.1038/s41598-020-60035-0

**Published:** 2020-02-21

**Authors:** Daniela Jara, Helia Bello-Toledo, Mariana Domínguez, Camila Cigarroa, Paulina Fernández, Luis Vergara, Mario Quezada-Aguiluz, Andrés Opazo-Capurro, Celia A. Lima, Gerardo González-Rocha

**Affiliations:** 1 Laboratorio de Investigación en Agentes Antibacterianos (LIAA), Departamento de Microbiología, Facultad de Ciencias Biológicas, Universidad de Concepción, Concepción, 4070386 Chile; 2 Programa Especial de Ciencia Antártica y Subantártica (PCAS), Universidad de Concepción, Concepción, 4070386 Chile; 3 Departamento de Ciencias Biológicas y Químicas, Facultad de Medicina y Ciencia, Universidad San Sebastián, Concepción, 4080871 Chile; 4 Millennium Nucleus for Collaborative Research on Bacterial Resistance (MICROB-R), Las Condes 12496, Lo Barnechea, Santiago 7690000 Chile; 5 Departamento de Medicina Interna, Facultad de Medicina, Universidad de Concepción, Concepción, 4070386 Chile; 6 Departamento Prevención y Salud Pública Odontológica, Facultad de Odontología, Universidad de Concepción, Concepción, 4070386 Chile

**Keywords:** Antimicrobial resistance, Environmental impact

## Abstract

Anthropic activity in Antarctica has been increasing considerably in recent years, which could have an important impact on the local microbiota affecting multiple features, including the bacterial resistome. As such, our study focused on determining the antibiotic-resistance patterns and antibiotic-resistance genes of bacteria recovered from freshwater samples collected in areas of Antarctica under different degrees of human influence. Aerobic heterotrophic bacteria were subjected to antibiotic susceptibility testing and PCR. The isolates collected from regions of high human intervention were resistant to several antibiotic groups, and were mainly associated with the presence of genes encoding aminoglycosides-modifying enzymes (AMEs) and extended-spectrum β-lactamases (ESBLs). Moreover, these isolates were resistant to synthetic and semi-synthetic drugs, in contrast with those recovered from zones with low human intervention, which resulted highly susceptible to antibiotics. On the other hand, we observed that zone A, under human influence, presented a higher richness and diversity of antibiotic-resistance genes (ARGs) in comparison with zones B and C, which have low human activity. Our results suggest that human activity has an impact on the local microbiota, in which strains recovered from zones under anthropic influence were considerably more resistant than those collected from remote regions.

## Introduction

The rise of antibiotic-resistant bacteria occurred few years after the beginning of the antibiotic era^1^, and is mediated either by mutations or by the horizontal transfer of foreign resistance genes among environmental and/or nosocomial bacteria. In this sense, it is well known that the environment can act as a reservoir of antibiotic-resistance genes (ARGs)^2–4^. Importantly, bacteria harboring AGRs can be disseminated to isolated regions and transfer these genes to endemic microorganisms^5,6^. Several factors related to this phenomenon have been described, in which anthropic activity and birds migration can mediate the dissemination of ARGs^7–11^. As such, the “One Health” initiative emerged as a global initiative oriented to generate a multidisciplinary approach to attain optimal health for humans, animals and the environment^12^. Accordingly, antibiotic-resistance is considered as an important threat to tackle under this new perspective.

Antarctica is considered the last pristine continent, due to its extreme weather conditions and geographical isolation^13^, which has allowed several ecosystems to be preserved almost unaltered. However, the presence of migrating animals and the increase in anthropogenic activity^14^, have favored the introduction of ARGs-harbouring bacteria^13,15^. Antibiotic-resistant isolates have been detected in both the South and North Poles, thus studies on the impact of human activity in these regions are highly needed in order to understand the effects of antibiotic-resistance beyond the clinical settings^4,16,17^. Due to the above, the aim of this study was to evaluate the antibiotic-resistance features of bacterial isolates recovered from freshwater samples collected in regions under differential anthropic influence in Fildes Peninsula, King George Island, Antarctica.

## Results

### Bacterial counts

Total counts of cultivable heterotrophic bacteria (CHB) from freshwater samples were 10^2^ to 10^3^ CFU/ml in zones A and B; whereas in zone C there were 10^1^ CFU/ml. There were no significant differences between the counts of CHB performed at 4°C and 12°C, which could be due to the psychrotolerant characteristic of the isolates. In the case of heterotrophic bacteria with decreased susceptibility to antibiotics, we observed significant differences (p < 0.05) between the counts from zones B and C in the plates supplemented with NAL, STR, KAN and CTX. Specifically, the highest counts of bacteria with decreased susceptibility to antibiotics were from zone B in agreement with the antibiotic susceptibility patterns, as a higher number of resistant isolates was also present in this region. On the other hand, it is important to remark that there were no significant differences between zones A and B regarding CHB with decreased susceptibility, which is congruent with the susceptibility profiles previously determined (p < 0.05).

Forty-eight isolates representing different colony morphotypes (with respect to mucous phenotype, colony morphology or size, and pigment production) were recovered from zone A (42 Gram-negative and 6 Gram-positive bacteria); twenty were recovered from zone B (all Gram-negative); and thirty-four from zone C (27 Gram-negative and 7 Gram-positive).

### Antibiotic resistance and ARGs

**Figure 1:**
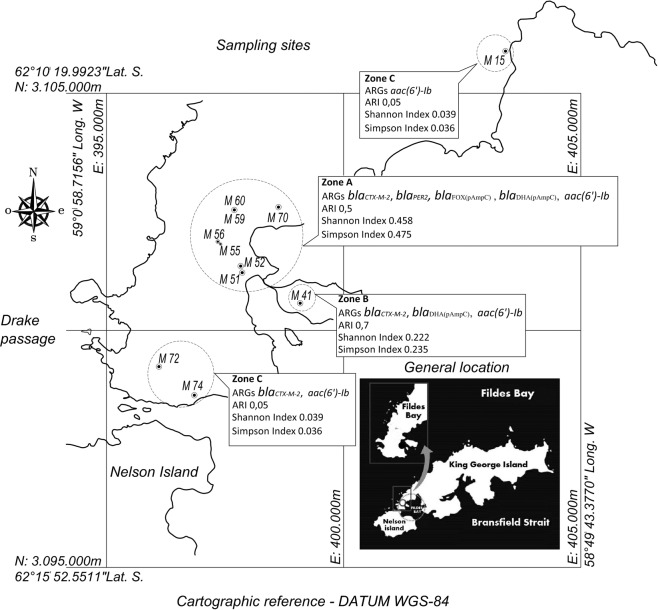
Sampling sites in Peninsula Fildes showing ARGs detected, antibiotic resistance index (ARI) and richness (Simpson Index) and diversity (Shannon Index) of genes in each area. Zone A: places under human influence M51, M52, M55, M56, M59, M60, M70; Zone B: places without human influence but with possible animal influence M41; and Zone C: remote places without human or animal intervention M15, M72, M74.

**Figure 2:**
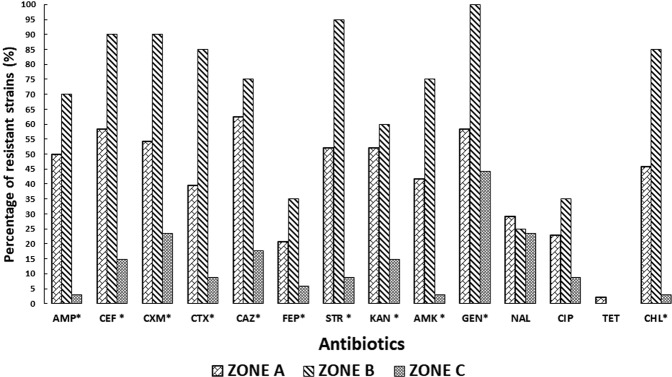
Percentage of antibiotic resistant strains in Antarctic areas. Antibiotics tested: ampicillin (AMP), cefalotin (CEF), cefuroxime (CXM), cefotaxime (CTX), ceftazidime (CAZ), cefepime (FEP), streptomycin (STR), kanamycin (KAN), amikacin (AMK), gentamicin (GEN), nalidixic acid (NAL), ciprofloxacin (CIP), tetracycline (TET), chloramphenicol (CHL). Antibiotics with p < 0.05 are indicated with (*).

Differences were observed between the bacteria recovered from zone A and zone B, where more resistant isolates were detected, in comparison with zone C, which was defined as a remote region with lower animal and human impact (Fig. 1). Therefore, a relationship can be established between the Antarctic zones sampled and the resistance to antibiotics (p < 0.05) (Fig. 2). Accordingly, zone B showed the highest percentages of antibiotic-resistant isolates. These isolates displayed resistance to β-lactams (mainly third-generation cephalosporins) and aminoglycosides. In addition, resistance to chloramphenicol, ciprofloxacin and trimethoprim was also observed.

In the case of zone A, the overall antibiotic-susceptibility patterns of the isolates were similar to zone B, but resistance to tetracycline and sulfamethoxazole was also observed. Resistance to β-lactams, aminoglycosides, ciprofloxacin and chloramphenicol was also observed in zone C.

Moreover, those isolates with inhibition zones ≤14 mm in diameter were screened for ARGs. Accordingly, in thirty-eight isolates from zone A, fifteen from zone B and seven from zone C the presence of 30 ARGs was investigated. The resistance to aminoglycosides was observed in the three zones studied, mediated by the presence of acetyltransferase-type AMEs, such as the *aac(6*′*)-Ib* gene, and resistance to beta-lactams in zones A and B was found to be due to the presence of extended-spectrum beta-lactamases (ESBL) and plasmid-mediated AmpC β-lactamases (Fig. 1). Zone A presented higher ARGs richness and diversity in comparison with zones B and C (Fig. 1). Interestingly, we determined that zone A was more dissimilar compared with zone C (Fig. 1), which could be due to the differences in anthropic activity. This could be indicating a distribution gradient of ARGs from zones under higher anthropic impact to less intervened regions.

### Bacterial identification

Thirty-nine isolates were selected for identification according to ARG diversity and colony morphotypes. The Biolog System, despite having a limited database, allowed us to identify four isolates: one from zone A and three from zone B. Molecular identification (sequencing of 16S rRNA gene) was performed on isolates that could not be identified by phenotypic characterization. Thus, it was possible to establish the following strain identification: From zone A, *Pseudomonas* sp. (n = 2), *P*. *veronii* (n = 1), *P. fluorescens* (n = 2), *Flavobacterium* sp. (n = 2) and *F. johnsoniae* (n = 1). From zone B, *Sphingobacterium thalpophilum* (n = 1), *Pseudomonas* sp. (n = 1), *P*. *fluorescens* (n = 1) and *P. tolaasii* (n = 1). Finally, *Janthinobacterium* sp. (n = 1) and *Hymenobacter* sp. (n = 1) were identified in zone C.

## Discussion

We quantified CHB recovered from freshwater samples in three zones of Antarctica, which are under different degrees of animal and human influence. The total counts of CHB were lower in zone C, which was defined as the less influenced area. These results are concordant with those published by Gonzalez-Rocha *et al*.^18^, in which they observed lower bacterial counts in remote zones in King George Island. The differences in bacterial counts could be attributed to the permanent presence of animals, such as migratory birds, in zone B. Settlements of migratory birds present in this zone could act as biological vectors of dissemination of antibiotic-resistant bacteria and ARGs from long distances^19^. Moreover, it is important to highlight that marine mammals also migrate long distances, increasing the probability of dissemination of these bacteria. Accordingly, resistant bacteria have been recovered from marine mammals and sharks in the west coast of the United States, of which 58% were resistant to at least one antibiotic, and 43% to more than one drug^20^. Despite these data, humans are more often associated to the dissemination of antibiotic-resistant bacteria. For instance, *Salmonella enterica* serovar Enteritidis related to human salmonellosis, has been detected in both Papua penguins (*Pygoscelis papua*) and Adelia penguins (*Pygoscelis adeliae*)^21^. Moreover, *Pasteurella multocida*, which is the etiological agent of avian cholera, has been detected in Rockhopper penguins (*Eudyptes chrysocome*)^22^, whereas other pathogenic bacteria such as *Clostridium cadaveris*, *C. sporogenes* and *Staphylococcus* sp. have been recovered from subcutaneous and muscular tissue of Adelia penguins^23^. Importantly, Antarctic migratory birds, such as skuas (*Catharacta skuas*) and seagulls (*Larus dominicanus*), whose habitats are under important anthropic influence, have been colonized by *Campylobacter jejuni* and *Yersinia* spp.^24^. On the other hand, we observed important differences in the antibiotic susceptibility patterns and in the bacterial richness and diversity of the ARGs detected among zones under human (zone A) and animal (Zone B) influence, in comparison with the more remote area (zone C). These differences could be due to the important influence of animals and humans that could be generating a selective pressure on the local microbiota^12^. It is also important to remark that the ARI indices, according to Krumperman^24^ showed differences between the zones, reflecting that the dissemination of the ARGs in the Antarctic environment could be influenced by the presence of both humans and animals. These results are in agreement with a previous report of ESBL-producing bacteria identified in freshwater samples collected in areas near the Bernardo O’Higgins (Antarctic Peninsula) and Arturo Prat (Greenwich Island) bases^25^. Even though the mechanisms of dissemination of ARGs in Antarctica are largely unknown, there is evidence that their spread is closely related to anthropogenic influence^26^ and to the presence of migratory animals^11,27,28^. Moreover, previous studies detected multidrug-resistant bacteria recovered from penguin feces in Torgensen Island and in the Palmer Station (Anvers Island)^15^. In addition we have previously published a study reporting *E. coli* resistant to STR and TET isolated from an area of Fildes Bay close to military and scientific bases^14^. In addition, Antelo and Batista (2013) detected bacterial isolates collected in Antarctica with high levels of antibiotic resistance, including aminoglycosides, β-lactams and trimethoprim, which is consistent with our findings^29^.

Interestingly, we detected isolates that were resistant to synthetic or semisynthetic antibiotics, such as SUL and TMP, in the zones with higher human activity, suggesting that both phenomena could be linked. While the data on antibiotic-resistance in Antarctic freshwater are scarce, a single report of *Enterococcus* sp. detected near Davis Station suggests that the discharge of insufficiently treated residual waters is introducing human pathogens that harbor ARGs into the Antarctic ecosystem^30^. The role of residual water is highly relevant since it is well known that resistant bacteria, ARGs and antibiotic debris can be disseminated through human feces. This was demonstrated by a study published by Karkman *et al*.^31^, in which the abundance of ARGs was correlated with fecal contamination and was not related to antibiotic selective pressure.

In the case of aminoglycosides resistance, we detected several AMEs, which could explain the resistant phenotypes observed among isolates. Our results revealed that aminoglycosides-acetylating enzymes were predominant among the resistant isolates. These enzymes have been previously identified in environmental isolates, in agreement with our results^4^. AMEs are normally plasmid-encoded, and also associated with transposons and integrons, which might contribute to their dissemination^32^. We screened for *aac(6)-Ib* and *acc(3)-IIa* genes, which account for resistance to KAN, TOB and AMK, and to GEN and TOB, respectively^33,34^. According to antibiotic-susceptibility patterns we detected resistance to GEN, STR, KAN and AMK in zones A, B and C. The presence of *aac(6*′*)-Ib* was detected in all areas and can explain the resistance to KAN and AMK. Interestingly, this gene has been commonly detected in Gram negative bacteria associated with humans, such as *E. coli* and *P. aeruginosa*^35^ and may represent a modification of the local resistome. A large number of genes can confer streptomycin resistance, including the phosphotransferase *aph(6)-Ia* gene (also named *strA*) and the *aph(6)-Id* gene (also named *strB*) which appear to be widely distributed in Gram-negative bacteria. *strA*-*strB* has been identified in bacteria circulating in humans, animals, and plants and these genes are frequently located on plasmids^36^.

Several β-lactamase genes were identified in our study; specifically, we detected the ESBL genes^37–40^ *bla*_CTX-M2_ and *bla*_PER-2_, and the plasmid-mediated AmpC β-lactamase genes *pAmp*_CDHA_, pAmp_CFOX_ in zone A, while *bla*_CTX-M2_ and *pAmp*_CDHA_ were identified in zone B. These enzymes mediate resistance to clinically relevant cephalosporins^41–43^, and were present in areas under human and wildlife influence. Interestingly, no β-lactamase genes were detected in zone C, where the collected isolates were considerably more susceptible to β-lactams. These findings suggest that these ARGs were introduced by either humans or animals into zones A and B. Our results are congruent with previous reports, in which ESBLs genes were detected in isolates collected in regions near scientific bases in Antarctica and native bacteria did not present any ARGs^26^.

According to the ARGs diversity analysis, we demonstrated that there is a gradient of richness and diversity from the less remote areas, where it is higher, to the more remote zones, reaffirming that ARGs are less prevalent in isolated regions. Similarly, Berglund^9^ demonstrated that ARGs and integrons were more prevalent in regions with anthropic activity, which includes the presence of residual water. Importantly, there is evidence that ARGs are present in the environment and are disseminated among bacteria^44^. Furthermore, it is important to remark that Antarctic bacteria are able to maintain and potentially disseminate ARGs, where it is possible that local microbiota could harbor naturally occurring ARGs, which could be potentially transmitted among bacteria^45^. It is difficult to measure the risk from the presence of antibiotic-resistant bacteria in this environment for both human and wildlife because there is a lack of data about the prevalence and persistence of ARGs in the environment^46^.

Even though more research is needed to achieve a better understanding of the dissemination routes of ARGs, our results suggest that human activity, together with migratory birds, could contribute to this phenomenon. These findings are illustrate the importance of the One Health approach, in which multi-disciplinary efforts are required to control the spread of ARGs and resistant bacteria among different environments^12^.

## Conclusions

Our findings show that the presence of antibiotic-resistance bacteria, and therefore ARGs, are more predominant in the zones of Fildes Peninsula that are more influenced by both humans and wildlife in comparison with remote areas. Moreover, it is very interesting to remark the presence of resistance to synthetic and semisynthetic antibiotics, which was identified in zones associated to human activity, suggesting that these resistant isolates could be linked with the presence of humans.

## Methods

### Sampling sites

Eleven freshwater samples were collected during the 49^th^ Antarctic Scientific Expedition (ECA49), January 2013. The samples were obtained from three areas: under human influence (zone A), animal influence (zone B) and areas with low animal and human influence (zone C), which are illustrated in Fig. 1. All the samples were transported on ice to the laboratory in Professor Julio Escudero Scientific Base (Chilean Antarctic Institute) and processed within 6 h from collection.

### Bacterial counts

Total counts of cultivable heterotrophic bacteria (CHB) were performed by the surface dissemination method in R_2_A agar (Merck, Darmstadt, Germany) supplemented with cycloheximide (50 µg/ml)^47,48^. Additionally, total counts of CHB with decreased susceptibility to antibiotics were carried out with the same methodology, but using plates supplemented with: nalidixic acid (NAL) (0.5 µg/mL), ciprofloxacin (CIP) (0.5 µg/mL), tetracycline (TET) (4 µg/mL), ampicillin (AMP) (4 µg/mL), cefotaxime (CTX) (0.5 µg/mL), kanamycin (KAN) (8 µg/mL), streptomycin (STR) (0.5 µg/mL), erythromycin (ERY) (4 µg/mL), sulfamethoxazole (SUL) (128 µg/mL), and trimethoprim (TMP) (4 µg/mL). The plates were incubated at 4°C during 15 days and at 15°C for 7 days. Different bacteria morphotypes were selected, according to their macroscopic and microscopic characteristics, and were preserved in a R_2_A broth with glycerol (50% v/v) at −80°C.

### Antibiotic susceptibility testing

Susceptibility tests were carried out by the disc diffusion method according to the CLSI guidelines^48^ using R_2_A as a replacement for Mueller-Hinton agar, except for TMP and SUL. The antibiotics tested were AMP (10 μg), CEF (30 μg), CXM (30 μg), CTX (30 µg), CAZ (30 μg), FEP (30 μg), STR (10 µg), KAN (30 µg), AMK (30 μg), GEN (10 μg), NAL (30 µg), CIP (5 µg), TET (30 µg) and chloramphenicol (CHL) (30 μg), and the plates were incubated at 15°C for 48 h. *Escherichia coli* ATCC 25922, *Staphylococcus aureus* ATCC 25923 and *Pseudomonas aeruginosa* ATCC 27853 strains were used as susceptibility controls. Inhibition areas ≤14 mm in diameter were considered as breakpoints to define resistance. The antibiotic resistance index (ARI) was determined according to Krumperman *et al*.^24^.

### Antibiotic resistance genes (ARGs)

Total bacterial DNA was extracted using the InstaGene matrix (Bio-Rad), according to the manufacturer’s instructions. ARGs were screened by conventional PCR using the primers listed in Table 1, covering diverse antibiotic groups.

**Table 1:** Oligonucleotides used in the detection of antibiotic resistance genes.

Gene	Primers	Nucleotide sequence (5′-3′)	Product size (bp)	Reference
16S rRNA	P0(16s) P6(16s)	GAGAGTTTGATCCTGGCTCAG CTACGGCTACCTTGTTACG	1400	^49^
*bla*_TEM_	TEMR TEMF	TGGGTGCACGAGTGGGTTAC TTATCCGCCTCCATCCAGTC	526	^52^
*bla*_SHV_	SHVR SHVF	CTGGGGAAACGGAACTGAAATG GGGGTATCCCGCAGATAAAT	389	^53^
*bla*_CTX-M-1_	m-CTX-MG1R m-CTX-MG1F	AAAAATCACTGCGCCAGTTC AGCTTATTCATCGCCACGTT	551	^54^
*bla*_CTX-M-2_	m-CTX-MG2R m-CTX-MG2F	CGACGCTACCCCTGCTATT CCAGCGTCAGATTTTTCAGG	742	^54^
*bla*_CTX-M-8_	m-CTX-MG8R m-CTX-MG8F	TCGCGTTAAGCGGATGATGC AACCCACGATGTGGGTAGC	923	^54^
*bla*_CTX-M-9_	m-CTX-MG9R m-CTX-MG9F	CAAAGAGAGTGCAACGGATG ATTGGAAAGCGTTACTCACC	803	^54^
*bla*_CTX-M-25_	m-CTX-MG25R m-CTX-MG25F	GCACGATGACATTCGGG AACCCACGATGTGGGTAGC	876	^54^
*bla*_MOX-1_, *bla*_MOX-2_, *bla*_CMY-1_, *bla*_CMY-8_ to *bla*_CMY-11_	MOXMR MOXMF	CAC ATT GAC ATA GGT GTG GTG C GCT GCT CAA GGA GCA CAG GAT	520	^55^
*bla*_LAT-1_ to *bla*_LAT-4_, *bla*_CMY-2_ to *bla*_CMY-7_, *bla*_BIL-1_	CITMF CITR	TGG CCA GAA CTG ACA GGC AAA TTT CTC CTG AAC GTG GCT GGC	462	^55^
*bla*_DHA-1_, *bla*_DHA-2_	DHAMF DHAMR	AAC TTT CAC AGG TGT GCT GGG T CCG TAC GCA TAC TGG CTT TGC	405	^55^
*bla*_ACC_	ACCMF ACCMR	AAC AGC CTC AGC AGC CGG TTA TTC GCC GCA ATC ATC CCT AGC	346	^55^
*bla*_MIR-1T_,* bla*_ACT-1_	EBCMF EBCMR	TCG GTA AAG CCG ATG TTG CGG CTT CCA CTG CGG CTG CCA GTT	302	^55^
*bla*_FOX-1_ to* bla*_FOX-5b_	FOXMR FOXMF	AAC ATG GGG TAT CAG GGA GAT G CAA AGC GCG TAA CCG GAT TGG	190	^55^
*bla*_PER-2_	PER-2 F PER-2REV	GTAGTATCAGCCCAATCCCC CCAATAAAGGCCGTCCATCA	738	^56^
*floR*	FloF FloR	AATCACGGGCCACGCTGTATC CGCCGTCATTCTTCACCTTC	215	^57^
*sul1*	Sul1F Sul1R	GTATTGCGCCGCTCTTAGAC CCGACTTCAGCTTTTGAAGG	408	^58^
*sul2*	Sul2F Sul2R	GAATAAATCGETCATCATTTTCGG CGAATTCTTGCGGTTTCTTTCAGC	810	^59^
*sul3*	Sul3F Sul3R	GAGCAAGATTTTTGGAATCG CATCTGCAGCTAACCTAGGGCTTTGGA	790	^60^
*drfA6*	dfrIb	GAGCAGCTICTITTIAAAGC TTAGCCCTTTIICCAATTTT	393	^61^
*drfA1*	D1 D2	ACGGATCCTGGCTGTTGGTTGGACGC CGGAATTCACCTTCCGGCTCGATGTC	257	^62^

### Species identification

Thirty-nine isolates harboring ARGs were selected for identification. They were initially run through the Biolog identification system (Biolog Inc.) using the MicroLog 1 software, following the manufacturer’s protocol. A probability >95% was set as threshold for species identification. Amplification and sequencing of 16S rRNA gene^49^ by conventional PCR using universal primers (Table 1) was performed on those isolates that could not be identified by the Biolog system. The sequences were compared against the National Center for Biotechnology Information (NCBI) nucleotide database using BLAST^50^.

### Statistical analyses

All statistical analyses were performed using the IBM SPSS Statistics software (v23.0, SPSS Inc®, Chicago, IL, United States). The Student’s t-test for independent samples was used to compare the mean values of the tested parameters for all the different temperatures. In addition, one-way ANOVA and the Tukey’s multiple range tests were applied in order to compare the values of the tested parameters for all the different sampling sites. The p-value <0.05 was established for the statistical significance.

Pearson’s Chi-square test was applied to identify associations between the origin of strain and antibiotic resistance. The p-value <0.05 was established for the statistical significance.

In order to compare the sampled zones in terms of richness and diversity of ARGs, we built a binary matrix (multidimensional scaling, MDS) utilizing the Primer 6 software package^51^. Specifically, both richness and diversity were calculated by the Shannon-Wiener and Simpson’s indices. Genetic similarity among the strains was determined by parametric dimensional scaling based on the Bray-Curtis coefficient.

## Data Availability

All data generated or analyzed during this study are included in this published article.
